# Bioactive Sphene-Based Ceramic Coatings on cpTi Substrates for Dental Implants: An In Vitro Study

**DOI:** 10.3390/ma11112234

**Published:** 2018-11-09

**Authors:** Hamada Elsayed, Giulia Brunello, Chiara Gardin, Letizia Ferroni, Denis Badocco, Paolo Pastore, Stefano Sivolella, Barbara Zavan, Lisa Biasetto

**Affiliations:** 1 Department of Industrial Engineering, University of Padova, Via F. Marzolo 9, 35131 Padova, Italy; hamada.elsayed@unipd.it; 2 Ceramics Department, National Research Centre, El-Bohous Street, 12622 Cairo, Egypt; 3 Department of Management and Engineering, University of Padova, Stradella San Nicola 3, 36100 Vicenza, Italy; giulia-bru@libero.it; 4 Department of Neurosciences, Section of Dentistry University of Padova, Via Giustiniani 2, 35128 Padova, Italy; stefano.sivolella@unipd.it; 5 Department of Biomedical Sciences, University of Padova, Via Ugo Bassi 58/B, 35131 Padova, Italy; chiara.gardin@unipd.it (C.G.); letizia.ferroni@unipd.it (L.F.); barbara.zavan@unipd.it (B.Z.); 6 Maria Cecilia Hospital, GVM Care & Research, 48033 Ravenna, Italy; 7 Department of Chemical Sciences, University of Padova, Via F. Marzolo 1, 35131 Padova, Italy; denis.badocco@unipd.it (D.B.); paolo.pastore@unipd.it (P.P.)

**Keywords:** implant, titanium, osseointegration, biocompatibility, bioactive ceramic coatings, sphene

## Abstract

Titanium implant surface modifications have been widely investigated to favor the process of osseointegration. The present work aimed to evaluate the effect of sphene (CaTiSiO_5_) biocoating, on titanium substrates, on the in vitro osteogenic differentiation of Human Adipose-Derived Stem Cells (hADSCs). Sphene bioceramic coatings were prepared using preceramic polymers and nano-sized active fillers and deposited by spray coating. Scanning Electron Microscopy (SEM) analysis, surface roughness measurements and X-ray diffraction analysis were performed. The chemical stability of the coatings in Tris-HCl solution was investigated. In vitro studies were performed by means of proliferation test of hADSCs seeded on coated and uncoated samples after 21 days. Methyl Thiazolyl-Tetrazolium (MTT) test and immunofluorescent staining with phalloidin confirmed the in vitro biocompatibility of both substrates. In vitro osteogenic differentiation of the cells was evaluated using Alizarin Red S staining and quantification assay and real-time PCR (Polymerase Chain Reaction). When hADSCs were cultured in the presence of Osteogenic Differentiation Medium, a significantly higher accumulation of calcium deposits onto the sphene-coated surfaces than on uncoated controls was detected. Osteogenic differentiation on both samples was confirmed by PCR. The proposed coating seems to be promising for dental and orthopedic implants, in terms of composition and deposition technology.

## 1. Introduction

Commercially pure titanium (cpTi) and its alloys represent the materials of choice for manufacturing orthopedic and dental implants because of their high mechanical properties, good corrosion resistance and excellent biocompatibility [1,2,3,4].

The successful outcome of the dental implants mainly depends on the osseointegration at bone-implant level. In recent years, several surface treatments have been applied to Ti implants with the aim of increasing the speed and success rate of osseointegration [5,6]. Surface modifications included acid etching, isothermal oxidation, hydrothermal synthesis method, and combination of sandblasting and acid etching (SLA) [5,6]. 

Bioactive coatings appear to be promising materials favoring a faster and more enhanced osseointegration [1]. Several different coatings were reported in the literature, including hydroxyapatite, bioglass, proteins, polysaccharides, drugs, calcium phosphate, and calcium silicate [7,8,9,10,11]. 

Hydroxyapatite (HA), an osseoconductive material, can induce direct formation of bone tissue around implants, thanks to its unique chemical composition similar to that of the inorganic mineral part of bone tissue [9,12]. Despite its excellent biological properties, there are concerns for using HA-coated Ti-6Al-4V implants. However, the mismatch of their thermal expansion coefficients may cause delamination at coating-substrate interface, compromising the long-term success of osseointegrated implants [13,14]. 

In addition, CaO–SiO_2_-based bioglasses have been investigated as coating materials. However, they have demonstrated poor bonding strength, due to the higher thermal expansion coefficient compared to that of Ti-6Al-4V [15,16], and high degradation rate [17,18,19,20,21].

Cp-Ti possess an average CTE (Coefficient of Thermal Expansion) of 8.9 (10^−6^ C^−1^), Ti alloys an average CTE of 9.2 (10^−6^ C^−1^). Hydroxyapatite shows CTE in the range between 10 and 14 (10^−6^ C^−1^), while sphene has a CTE of 6 (10^−6^ C^−1^). SiO_2_-CaO bioactive glasses show CTE ranging between 8.5 and 19 (10^−6^ C^−1^).

However, the clinical applications of bioglasses are limited by their low bending strength, high brittleness, low fracture toughness and workability. To overcome the major drawbacks related to the use of bioglass, several silicate-based ceramics were produced and tested as bioactive materials for bone tissue regeneration. Moreover, when compared to CaSiO_3_ ceramics and tricalcium phosphate TCP (TriCalcium Phosphate), sphene (CaTiSiO_5_) ceramics drove higher bone-derived cell attachment, spreading, proliferation and differentiation. CaTiSiO_5_ ceramics have shown significantly higher chemical stability. Also, they have shown better chemical and biological properties compared to HA ceramics. 

Sphene (CaTiSiO_5_) bioactive coatings on titanium substrates were produced using various methods, such as plasma-spraying [14], sol-gel method [22], a hybrid technique of microarc oxidation coupled with heat treatment [23], and airbrush spray coating [24]. These studies have proved their chemical stability, excellent bonding strength (improved compared to HA coatings), bioactivity and cellular biocompatibility. Taken together, these results suggest that sphene ceramics may be potential candidates for bioactive coatings for orthopedic and dental implants. This reduced difference between CTE of cpTi and CaTiSiO_5_ compared to that of cpTi and HA, is known to be the main property responsible for their improved adhesion [14,22,23,24,25,26].

Recently, our research group deposited sphene coatings from a preceramic polymer precursor “Silicone” onto Ti plates by the airbrush spray technique. Coatings exhibited excellent adhesion to the substrates and homogeneous deposition mode [24,27]. 

The novelty of this procedure compared to those reported in the literature consists both of the development of sphene synthesis via preceramic polymers route and nano-sized precursors, as well as in the deposition technique. The main advantages of the proposed technology can be summarized as follow:

- The use of preceramic polymer together with nano-sized precursors allowed for the improvement of reaction efficiency to produce sphene as well as an optimized suspension useful for spray coating via airbrush.
- The use of spray coating is a low-cost technique capable of producing coatings possessing a controlled morphology (roughness and thickness) and thereby improved adhesion to the metallic substrate.
- Compared to high temperature coating techniques, such as plasma coating, the spray coating used in this work is performed at room temperature and further heating of the samples at relatively low temperature.
- The optimized composition of the bioceramic coating together with its improved adhesion to the substrate is the result of the synergic effect of synthesis of sphene via preceramic polymer route and the deposition technique we used.

The primary objective of the current research consists of evaluating the effect of polymer-derived sphene-coated Ti substrates on the osteogenic differentiation of Adipose-Derived Stem Cells (ADSCs) in vitro. The secondary aim consists of assessing sphene coating composition, its in vitro chemical stability, and its micrometer-scale profile- and areal-topography.

## 2. Materials and Methods

### 2.1. Samples Preparation and Coating Deposition

Sphene (CaTiSiO_5_) bioactive ceramic coating on Titanium plates (cpTi) was developed by using a preceramic polymer “silicone” approach as previously reported [24,27]. Preceramic silicone “polymethylsiloxane” (commercially available and known as “SILRES^®^ MK”, Wacker-Chemie GmbH, München, Germany), isopropanol, nano-sized CaCO_3_ powder (PlasmaChem, Berlin, Germany, 90 nm) and nano-sized TiO_2_ powders (Evonik Degussa GmbH, Essen, Germany, 21 nm) were used as starting materials. 

Briefly, to prepare the coating suspension, SILRES^®^ MK silicone resin powder was used as SiO_2_ precursor. The MK silicone was placed in isopropyl alcohol. Active fillers of nanoparticles of CaCO_3_, and TiO_2_ were mixed with the MK silicone under magnetic stirring. The total solid content load in the suspension was ~48 vol%. The addition of active fillers was in stoichiometric molar ratio that allows developing the sphene bioceramic as a final ceramic product after sintering at relative lower temperature (i.e., 950 °C). 

The suspension was homogenized by sonication for 15 min and then transferred into an automatic airbrush (Prona-RA-C2, PronaTools, Toronto, Canada M3J 3A1) for spray coating deposition. During the coating process, the silicone-fillers mixture was kept homogenous by magnetic stirring. 

Rectangular plates of 13 × 13 × 3 mm^3^ size of commercially pure Ti grade 2 (Torresin Titanio s.r.l, Padova, Italy) were used as substrates. The chemical composition of the cpTi plates, as given by the producer (wt.%), was: Fe 0.060, O 0.140, N 0.004, H 0.003, C 0.016, Ti 99.78. Before coating deposition, the cpTi plates were ultrasonically cleaned with acetone, alcohol, and deionized water for ten minutes for each passage, and finally the titanium substrates were dried using compressed air. The use of cpTi instead of Ti alloys as substrate was driven by the choice of restricting the field of study to dental implants. 

After many preliminary tests, the processing setup was optimized. The distance between the substrate and nozzle distance was fixed to be 350 mm. The diameter of nozzle of the airbrush was of 1 mm. The air inlet was set at three bars and a deposition time of 1 s was chosen. After deposition process, the plates were left to dry in air at room temperature. Then, the coated samples were heat-treated in static air at 950 °C for 1 h (using 5 °C/min as heating rate).

### 2.2. Surface and Coating Characterization 

The morphology of the surface of both uncoated (cpTi) and sphene-coated (Sphene) cpTi plates was investigated by Field Emission Gun Scanning Electron Microscopy (FEG-SEM, Quanta 250 Fei, Eindhoven, The Netherlands). 

X-ray diffraction (XRD) patterns of the coatings, after thermal treatment, were collected with an X-ray diffractometer (XRD Bruker D8 Advance, Milano, Italy) operated with Cu-Kα radiation 40 mA and 40 mV. The Rietveld refinement was subsequently applied to quantitatively analyze the XRD data using MAUD (Materials Analysis Using Diffraction) software and COD (Crystallography Open Database).

Surface topography was investigated using a stylus profilometer (Form Talysurf i-Series, Taylor Hobson Ltd., Leicester, UK), a high-resolution system for both profile- and areal-topography measurements. For 2D profile measurements (R_a_ and R_z_), two plates of each group (cpTi and Sphene) were analyzed and a total of 3 scans (evaluation length equal to 5 mm) were extracted from the surface of each sample. For 3D areal measurements, at least 4 squared areas of 0.5 mm × 0.5 mm, extracted from the same surface, in at least two samples per group (cpTi and Sphene) were examined by 3D scanning. Areal parameter S_a_ and S_z_ were selected to describe the surface topography. Profile and areal data were filtered to eliminate waviness as well as form components of surface topography by applying a Gaussian filter with a sampling length equal to 0.8 mm, in accordance with ISO 4288 and ISO 25178. Data evaluation was performed with Talymap analysis software (Taylor Hobson Ltd., Leicester, UK).

### 2.3. Chemical Stability

The chemical stability of the sphene-coated cpTi substrates was evaluated in Tris-HCl buffer solution. The coated samples were immersed in the buffer solution of tris-(hydroxymethyl)-aminomethane (Tris, (CH_2_OH)_3_CNH_2_) and hydrochloric acid (HCl) with a pH value of 7.4 and were examined at 1, 3 and 7 days. The ratio of plate surface area to Tris-HCl solution volume was 0.1 cm^2^/mL. 

Before and after soakings, dried coated samples were analyzed by means of a stylus profilometer (Form Talysurf i-Series) to evaluate changes in surface roughness. The roughness values of 3 different tracks (5 mm evaluation length) and 4 square areas (0.5 mm × 0.5 mm) per sample were recorded. The following profile roughness parameters, R_a_ and R_z_, and areal parameters, S_a_ and S_z_, were selected. 

The variation of Ca, Si and Ti concentrations in the Tris-HCl solution was measured by Inductively Coupled Plasma Optical Emission Spectrometry (ICP-OES; iCAP 7400 ICP-OES, Thermo Fisher Scientific, Cambridge, UK) and confirmed by Inductively Coupled Plasma Mass Spectrometry (ICP-MS, Agilent Technologies 7700 × ICP-MS system, Agilent Technologies International Japan, Ltd., Tokyo, Japan). The ICP-MS was tuned daily using a tuning solution containing 10 μg L^−1 140^Ce, ^7^Li, ^205^Tl, and ^89^Y (Agilent Technologies, UK). A 100 μg L^−1^ solution of ^45^Sc and ^115^In (Aristar, BDH, UK) prepared in HNO_3_ 1.4% was used as internal standard through addition to the sample solution via a T-junction. 

All calibrations of Ca, Si and Ti were obtained in Tris-HCl solution using the multi-standard CCS-5 (Inorganic-Ventures) for Si and Ti (100 mg L^−1^), and multi-standard IMS-120 (Ultra Scientific Multistandard) for Ca (1000 mg L^−1^). All regressions were linear with a determination coefficient (R^2^) larger than 0.999. Five samples for each time point were taken and measured five times. Data were given as mean ± standard deviation. The dissolution kinetics of Ca, Si and Ti were obtained from the released concentrations.

### 2.4. Human ADSCs Isolation and Cell Culture 

Human ADSCs (hADSCs) were isolated from the adipose tissue of healthy patients undergoing cosmetic surgery procedures. Tissue collection protocol received a favorable ethical opinion by the Local Bioethical Committee, Padova University and all participants provided written consent. In addition, all experiments performed with human-derived materials were conducted in accordance to the relevant guidelines and regulations. The tissues were digested, and the cells isolated and expanded as described in Gardin et al. [28].

### 2.5. Seeding of hADSCs 

hADSCs were seeded on both uncoated and sphene-coated samples, in a 12-well plate, with a density of 2 × 10^4^ cells/sample. The cells were cultured in DMEM (Dulbecco’s Modified Eagle’s Medium) High Glucose or Osteogenic Differentiation Medium, at 37 °C and 5% CO_2_, for 21 days. Osteogenic Differentiation Medium was made of DMEM High Glucose supplemented with 10 nM dexamethasone, 10 mM b-glycerophosphate, and 10 ng mL^−1^ of basic fibroblast growth factor. Both media were completed with 10% fetal bovine serum and 1% penicillin/streptomycin. After 21 days of culture, Methyl Thiazolyl-Tetrazolium (MTT) test, Scanning Electron Microscopy (SEM) analysis, immunofluorescent staining with phalloidin, Alizarin Red S staining and quantification, and real-time PCR were carried out.

### 2.6. MTT Assay

To assess the proliferation rate of hADSCs grown on both uncoated and coated samples, a colorimetric mitochondrial viability assay was performed as described by Denizot and Lang [29] with minor modifications. After removing the culture medium, the samples were incubated in 1 mL of 0.5 mg/mL MTT solution in phosphate buffered saline (PBS) for 3 h at 37 °C. The MTT solution was then removed, and each sample was extracted with 0.5 mL of 10% dimethyl sulfoxide in isopropanol for 30 min at 37 °C. For each sample, Optical Density (O.D.) values, at 570 nm, were recorded in duplicate on 200 μL aliquots using a multilabel plate reader (Victor 3, Perkin Elmer, Milan, Italy).

### 2.7. SEM Analysis

For SEM imaging, hADSCs grown on both uncoated and sphene-coated samples for 21 days were fixed in 2.5% glutaraldehyde in 0.1 M cacodylate buffer for 1 h, then progressively dehydrated in ethanol. Cell spreading and morphology were evaluated using FEG-SEM (Quanta 250 Fei, Eindhoven, The Netherlands). 

### 2.8. Immunofluorescence

Cell adhesion to the scaffolds surface was evaluated by immunofluorescent staining with phalloidin. Briefly, cells were fixed in 4% paraformaldehyde in PBS for 10 min, then permeabilized with 0.1% triton X-100 in PBS for 30 min at room temperature. 5 mg/mL phalloidin were then used for fluorescent staining of actin filaments, whereas nuclear staining was performed with 2 μg mL^−1^ Hoechst H33342 solution for 5 min. Image acquisition was obtained with an inverted optical microscope DMI4000 B (Leica Microsystems, Wetzlar, Germany).

### 2.9. Alizarin Red S Staining and Quantification

The formation of extracellular mineral deposits onto uncoated and sphene-coated samples was detected by Alizarin Red S staining and quantification. Cells were fixed in 4% paraformaldehyde in PBS for 10 min at room temperature. Then, cells were stained adding 0.5 mL of 40 mM freshly prepared Alizarin Red S solution (pH 4.2) for 20 min at room temperature. After washing with double-distilled water, the Alizarin Red S stained areas were extracted with 0.5 mL of 10% cetylpyridinium chloride solution for 1 h at room temperature under gentle agitation. For each sample, O.D. values at 570 nm were recorded in duplicate on 200 μL aliquots using a Victor 3 plate reader.

### 2.10. Real-Time PCR

Total RNA was extracted from hADSCs, cultured on both uncoated and sphene-coated samples for 21 days, with a Total RNA Purification Plus Kit (Norgen Biotek Corporation, Thorold, ON, Canada). For the first-strand cDNA synthesis, 1 µg of total RNA of each sample was reverse transcribed with a SensiFAST™ cDNA Synthesis Kit (Bioline, London, UK), following the manufacturer’s protocol. Human primers were selected for each target gene using a Primer 3 software. Real-time PCRs were run using the chosen primers at a concentration of 400 nM and SensiFAST™ SYBR No-ROX Kit (Bioline) on a Rotor-Gene 3000 (Corbett Research, Sydney, Australia). The thermal cycling conditions were as follow: 2 min denaturation at 95 °C; 40 cycles of 5 s denaturation at 95 °C; annealing for 10 s at 60 °C; and 20 s elongation at 72 °C. Differences in gene expression were calculated by normalizing to the expression of the Transferrin Receptor (TFRC) housekeeping gene.

### 2.11. Statistical Analysis

One-way analysis of variance (ANOVA) was used for data analysis. *t*-tests were used to determine data significance (*p* < 0.05). All tests were performed using SPSS 16.0 software (SPSS Inc., Chicago, IL, USA) (licensed to the University of Padua, Padova, Italy).

## 3. Results

### 3.1. Surface Characterization 

**Figure 1:**
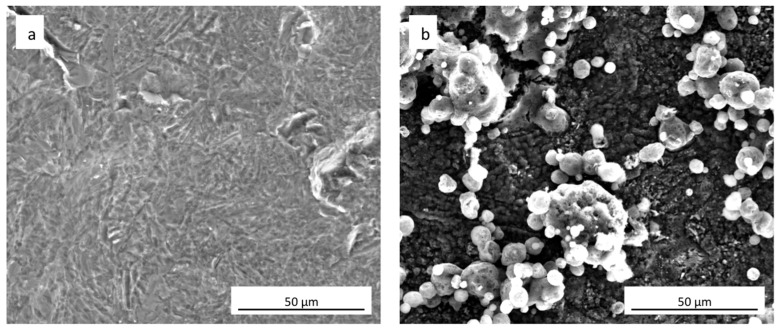
Surface morphology (SEM-FEG) of (**a**) as-received uncoated cpTi and (**b**) sphene-coated cpTi.

FEG-SEM images of uncoated cpTi and sphene-coated surfaces are shown in Figure 1a,b, respectively. Sphene coating is characterized by a homogenous grey substrate, composed of tetragonal crystals <1 μm, and “white islands” growing on it. These white agglomerates appear to be composed of vertically aggregated spherical particles. As reported in previous work [27], EDS (Energy Dispersive X-ray Spectrometry) analysis identified Ti, O, Si and Ca in areas corresponding to these white spherical structures. Instead, in the grey phase Ti and O were mainly present.

**Figure 2:**
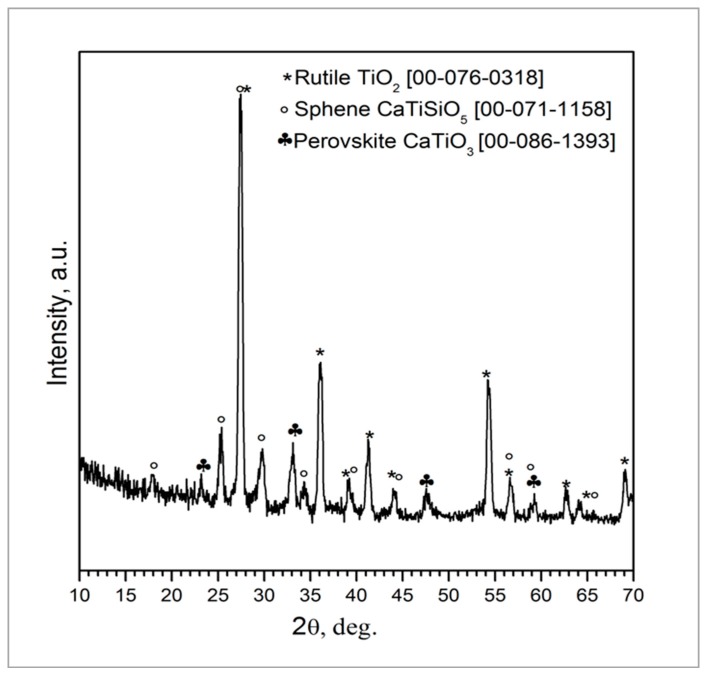
XRD pattern of sphene-coated cpTi after heat treatment at 950 °C in air for 1 h.

The coating composition was further investigated by XRD analysis and Rietveld refinement (Figure 2) that showed the presence of rutile (TiO_2_, 60 wt.%) followed by sphene (CaTiSiO_5_, 31 wt.%) and perovskite (CaTiO_3_, 9 wt.%). The as-received TiO_2_ powders, used as a filler to develop the sphene ceramic, are characterized by a weight percentage ratio of 80 to 20 between anatase and rutile, as claimed in the manufacturer’s datasheet. Anatase and rutile are the two main polymorphs of titanium oxide. They both exhibit tetragonal crystalline structure but obey different space groups. Anatase is a metastable phase, and the transformation to the stable rutile structure occurs as irreversible phase transformation in the range between 600 °C and 700 °C [30]. The treatment at 950 °C, performed in air, may have induced the nucleation and growth of rutile crystals before sphene synthesis, to yield a final weight percentage ratio of 60 wt. % rutile. The higher stability of rutile compared to anatase may have inhibited the reaction to produce sphene at this temperature. It has indeed been reported in the literature that the formation of sphene by the sol-gel method is complete at 1300 °C [31]. As previously reported [24], the choice of keeping the temperature at 950 °C was driven by the need for preserving the structure of the cpTi plate without affecting the bonding of the coating to the substrate. Further studies are ongoing aimed at increasing the amount of sphene produced by the preceramic polymer precursor route.

**Figure 3:**
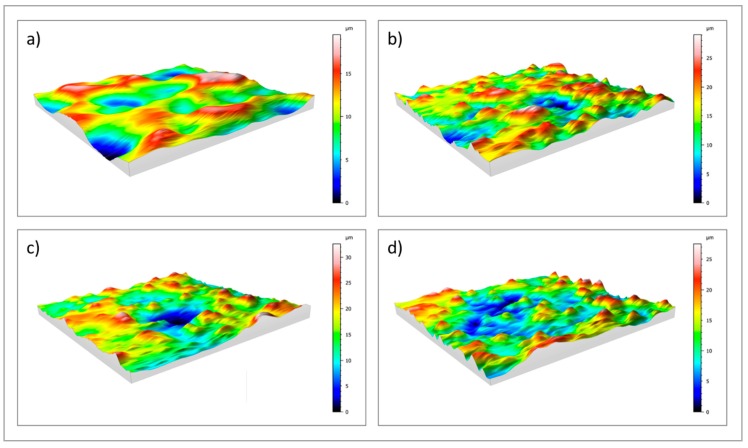
3D maps of: (**a**) uncoated cpTi; (**b**) sphene-coated cpTi; (**c**) sphene-coated cpTi after 1 day in Tris-HCl buffer solution; (**d**) sphene-coated cpTi after 7 days in Tris-HCl buffer solution.

Surface roughness was investigated by means of a profilometer. 2D profile measurements (R_a_ and R_z_) and 3D areal measurements (S_a_ and S_z_) of uncoated (cpTi) and sphene-coated (Sphene) cpTi substrates are reported in Table 1 and Figure 3. 

A clear difference in the values of R_a_ and R_z_ between the cpTi substrate and the sphene-coated substrate can be observed in the 2D measurements. However, this difference is smoothed in the 3D areal measurements. Indeed, the surface of the cpTi substrate is characterized by a flatter morphology (Figure 3a) than the sphene-coated sample (Figure 3b). The sphene coating presents a high number of peaks homogeneously distributed, while the cpTi substrate presents a limited number of peaks but characterized by a wider area. The yellow and red peaks in Figure 3b well correspond to the edges of the white agglomerates detected by SEM observations (Figure 1b).

### 3.2. Chemical Stability

The 2D roughness data after Tris-HCl immersion test show a slight increase after 1 day of soaking time. However, this increase in roughness values shows an almost constant trend after 3 and 7 days. In accordance to these data, 3D maps of sphene after immersion in Tris-HCl solution (Figure 3c,d) demonstrated a reduction in the numbers of peaks after the dissolution test, with a similar pattern of peak and valley distributions at 1, 3 and 7 days after soaking. This is confirmed by S_a_ and S_z_ values that were reported in Table 1.

**Figure 4:**
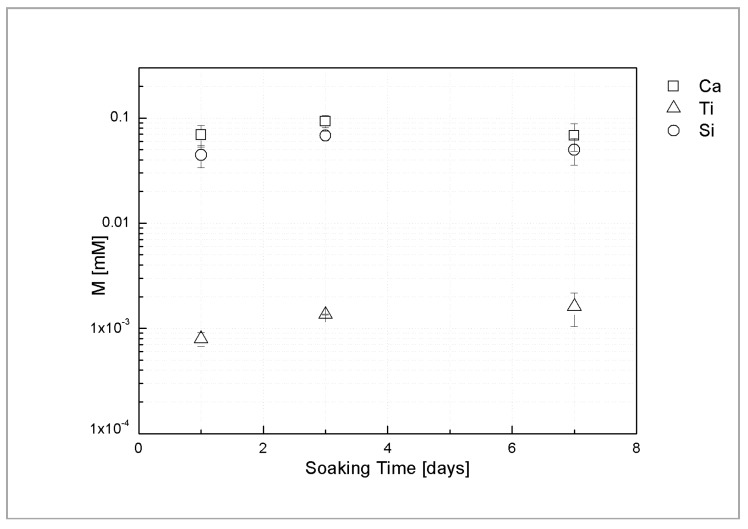
Ca, Ti and Si ion release of sphene-coated samples, after 1, 3, and 7 days of soaking time.

Figure 4 shows ICP analysis of Ca, Si and Ti ion release of the sphene coating in Tris-HCl solution at different time points. The average concentrations of Ca and Si ions, after 1 day of soaking, are 0.069 mM and 0.045 mM, respectively. Similar concentration levels were found after 3 and 7 days of immersion in Tris-HCl solution. Only trace amount of Ti ions released from the coating was detected (0.001–0.002 mM).

These findings are consistent with the above mentioned areal-topography measurements and suggest that the dissolution occurs only in the first hours after soaking. 

### 3.3. Cell Proliferation

**Figure 5:**
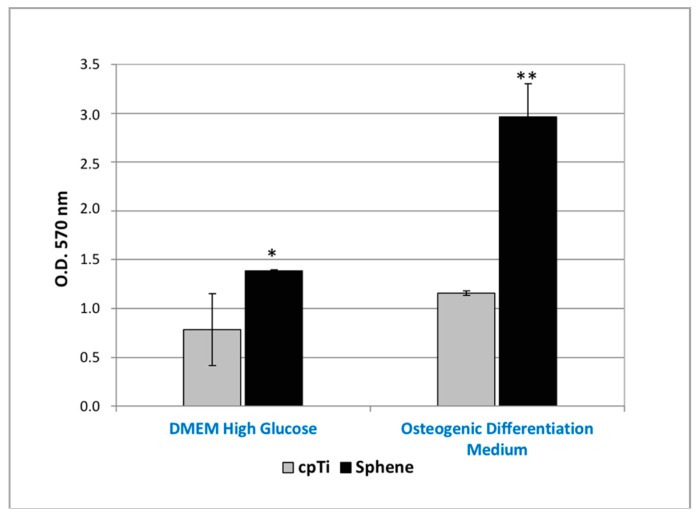
MTT assay of hADSCs cultured for 21 days on uncoated cpTi samples (*cpTi*) and on sphene-coated (*Sphene*) samples in DMEM High Glucose or Osteogenic Differentiation Medium. * *p* < 0.05, ** *p* < 0.01, *** *p* < 0.001.

The MTT assay proved that hADSCs were able to proliferate on both sphene-coated and uncoated samples (Figure 5). The O.D. values recorded for cells loaded on coated samples were found to be significantly (*p* < 0.05) higher to those observed for the controls in both the experimental conditions. Moreover, from MTT results, it was shown that hADSCs’ proliferation was higher when hADSCs were cultured in Osteogenic Differentiation Medium compared to in DMEM High Glucose, in particular for the sphene-coated samples.

### 3.4. Cell Adhesion and Morphology

**Figure 6:**
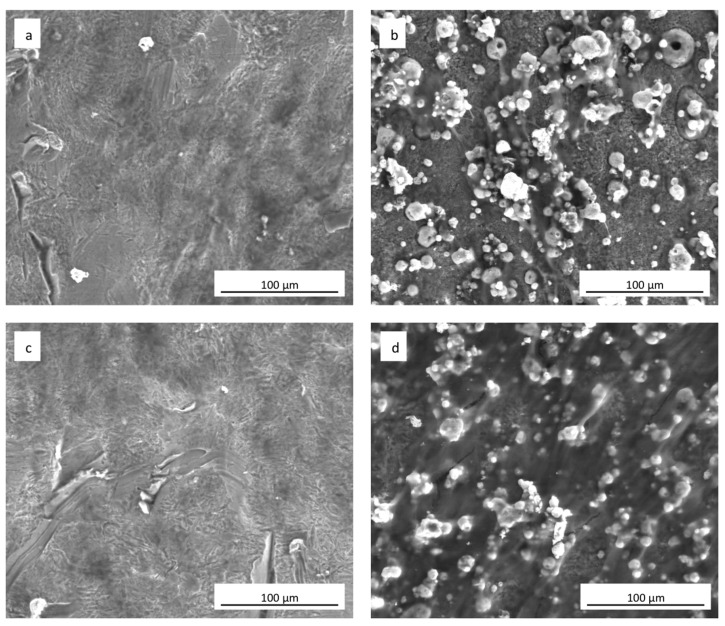
SEM images (1000× magnification) of hADSCs grown for 21 days on (**a**) uncoated cpTi in DMEM High Glucose; (**b**) sphene-coated cpTi in DMEM High Glucose; (**c**) uncoated cpTi in Osteogenic Differentiation Medium; (**d**) sphene-coated cpTi in Osteogenic Differentiation Medium.

The SEM analyses showed how hADSCs anchored to the surface of the specimens (Figure 6a–d). The cells were extremely flat with the typical star morphology associated with the osteoblastic-like phenotype and their distribution was similar, independent of the culture medium used (Figure 6a,c). After 21 days of culture onto the sphene-coated surfaces, cells showed short and thin filopodia when grown in DMEM High Glucose (Figure 6b); whereas they appeared flat and overlapped when cultured in the presence of the Osteogenic Differentiation Medium (Figure 6d). In both cases, the sphene coating remained intact during the culturing time, thus demonstrating its chemical stability.

### 3.5. Cytoskeletal Organization

**Figure 7:**
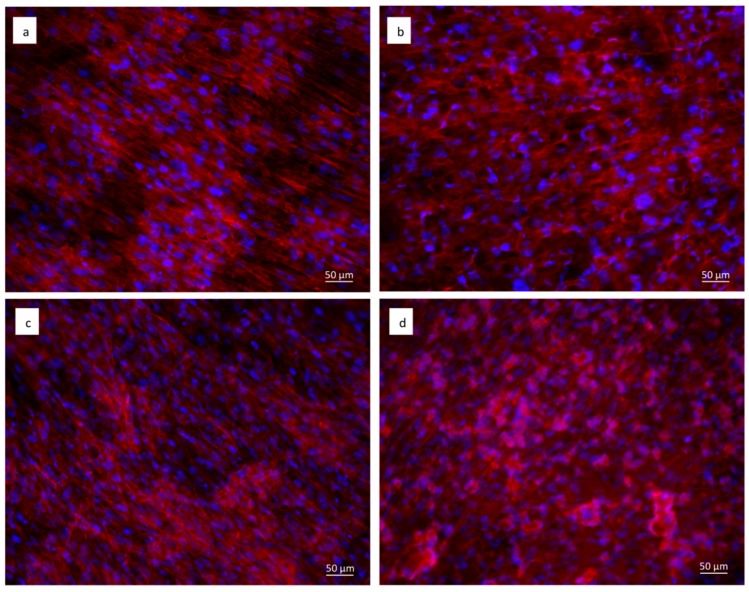
Immunofluorescent staining of the actin filaments with phalloidin (in red). Cell nuclei are counterstained with Hoechst (in blue). hADSCs seeded for 21 days on: (**a**) uncoated cpTi in DMEM High Glucose; (**b**) sphene-coated cpTi in DMEM High Glucose; (**c**) uncoated cpTi in Osteogenic Differentiation Medium; (**d**) sphene-coated cpTi in Osteogenic Differentiation Medium.

Staining with fluorescent phalloidin showed that cells were able to attach and spread on both the cpTi (Figure 7a,c) and sphene-coated samples (Figure 7b,d). After 21 days of culture, hADSCs had completely colonized the surfaces and formed a continuous layer with no significant differences between the experimental conditions. In vivo, such as in periosteum, osteoblasts are aligned with the collagen they produce and also on system cells are aligned to the collagen substrate. Cytoskeletal stresses and tension increase with increasing ECM (ExtraCellular Matrix) stiffness. The cytoskeleton provides a structural frame for the cell through Focal adhesions. Focal adhesion forms when adapter proteins link the cytoskeleton to integrins, which permits cells to adhere to the substrate. A variety of signaling proteins are also associated with focal adhesions, including focal adhesion kinase (FAK), an important mediator of signaling at these centers. Forces are also transmitted to the substrate at these sites. Osteogenic differentiation can also be affected by the substrate properties where cells are seeded. On our samples, we can confirm that the shape of a cell follows the steps that occur in vivo.

### 3.6. In Vitro hADSC Osteogenic Differentiation

**Figure 8:**
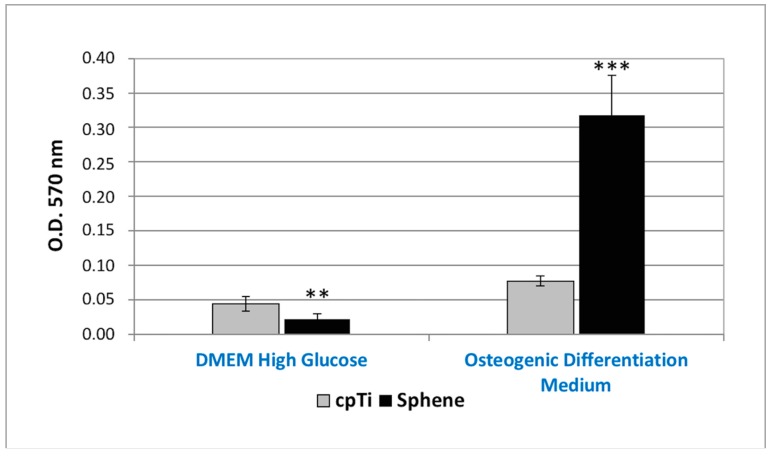
Alizarin Red S quantification of calcium deposits produced by hADSCs seeded onto *cpTi* or *Sphene* surfaces in DMEM High Glucose or Osteogenic Differentiation Medium for 21 days. * *p* < 0.05, ** *p* < 0.01, *** *p* < 0.001.

The in vitro osteogenic differentiation of hADSCs was first evaluated by marking of extracellular calcium deposition with Alizarin Red S staining and quantification at 21 days of culture (Figure 8). A difference in the presence of calcium deposits on the sphene-coated surfaces and the cpTi controls was evidenced for hADSCs cultured in the Osteogenic Differentiation Medium. Calcium deposits were significantly (*p* < 0.05) higher in the first experimental condition. The accumulation of calcium deposits by hADSCs was not observed when cells were seeded on the sphene-coated or uncoated samples in DMEM High Glucose.

### 3.7. Real-Time PCR

Real-time PCR was used to quantify the relative expression level of several osteogenic differentiation markers. In particular, expression of runt-related transcription factor 2 (RUNX2), osterix (OSX), collagen type I (COL1A1), osteocalcin (OC), osteonectin (ON), osteopontin (OPN), alkaline phosphatase (ALPL), and receptor activator of nuclear factor kappa-B ligand (RANKL) has been investigated. Runx2 and Osterix are the main transcription factors involved on osteoblastic commitment. Collagen is the principal component of the bone matrix. Alkaline Phosphatase is involved on the calcification of the Extracellular matrix. 

Osteonectin is a protein which role is related to the connection between extracellular matrix component and osteoblast. It is mostly produced during the first phases of bone regeneration or during bone remodeling [32,33,34]. 

**Figure 9:**
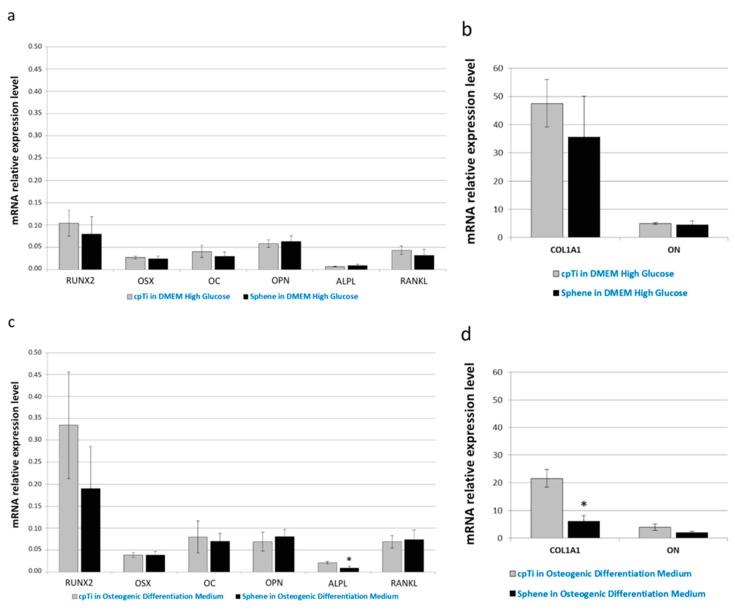
Real-time PCR analysis of the osteogenic differentiation markers runt-related transcription factor 2 (RUNX2), osterix (OSX), collagen type I (COL1A1), osteocalcin (OC), osteonectin (ON), osteopontin (OPN), alkaline phosphatase (ALPL), and receptor activator of nuclear factor kappa-B ligand (RANKL). Data are normalized to the expression of the transferrin receptor (TFRC) internal reference. Gray bars indicate the relative expression level of the selected genes in the hADSCs seeded onto *cpTi* samples in DMEM High Glucose (**a**,**b**) or Osteogenic Differentiation Medium (**c**,**d**) for 21 days. Black bars represent the gene expression level of the same markers in the hADSCs seeded onto *Sphene* samples in DMEM High Glucose (**a**,**b**) or Osteogenic Differentiation Medium (**c**,**d**) for 21 days.

As evident in Figure 9a,c, the relative expression of RUNX2, OSX, OC, OPN, and RANKL did not change significantly between the uncoated and sphene-coated surfaces, both when cells were grown in DMEM High Glucose or in Osteogenic Differentiation Medium. Similarly, there was no noticeable difference in ALPL (Figure 9a), COL1A1 and ON (Figure 9b) mRNA expression between cpTi and sphene-coated samples in DMEM High Glucose; instead, a slight down-regulation of ALPL (Figure 9c) and COL1A1 (Figure 9d) expression was identified in cells seeded onto the sphene surfaces in the presence of Osteogenic Differentiation Medium.

## 4. Discussion

In the present study, the sphene-based coatings on cpTi substrates showed a rough morphology, with roughness Ra in the range of 3.25 to 4.75 μm. The morphology was characterized by small crystals made of TiO_2_ and CaTiO_3_ (valley of Figure 4) and islands of unregular shape that were detected by EDS analysis to be composed of sphene [27]. The quantitative XRD analysis performed on the coated substrate showed the reaction efficiency and the amount of sphene that was about 31 wt. %. 

Micro-roughened surfaces were demonstrated to have a positive effect on early blood cell/implant interactions [35] and osteoblast proliferation [36]. In addition, surface topography was found to influence osteoconduction in both animal studies [37,38] and human retrieval study [39], with good results shown by rough implant surfaces. Sphene coatings examined in the present study possessed a moderately rough surface. The surface roughness quantified by 3D areal measurements showed a good correspondence with 2D measurements in both cpTi and coated cpTi. 

Even though the release of Ca and Si ions has been demonstrated to favor osteoblastic proliferation and differentiation [18,19,40], one of the major drawbacks of biosilicate ceramic coatings, such as CaSiO_3_ coatings, consists of their poor chemical stability and high degradation rate that might compromise their long-term stability [41,42]. Therefore, a limited release of these ions would enhance coating bioactivity and, on the other hand, would not interfere with the integrity of the coating itself [43]. 

To assess the chemical stability of the coatings, Tris-HCl was chosen due to the absence of Ca, Si or Ti ions in its composition. The results of dissolution tests in Tris-HCl showed that the dissolution happened only during the first hours of soaking and then remained constant after 1, 3 and 7 days. This behavior agrees with the results reported by Wu et al. [22], thus proving that the developed coating is comparable in terms of chemical properties to standard HA. At the same experimental conditions of the present study, a lower released Ca concentration (2.74 ppm) after 7 days of soaking in Tris-HCl was found in the sphene-coated samples here investigated as compared to the values of around 20 ppm and >110 ppm of plasma-sprayed sphene ceramic coatings and HA coatings, respectively [14]. Moreover, as found by Wang et al. [43], a higher concentration of Ca ions released to the solution was measured compared with the concentration of Si ions released after 7 days of immersion. In addition, only an irrelevant amount of Ti ions was detected by Wang et al. [43] in the Tris-HCl after one week of soaking. 

The roughness of samples, after dissolution tests slightly increased compared to the starting samples. This is mainly observable in the areal measurements where a sharp increase of Sz was measured from unetched sphene to 1-day soaking time in Tris-HCl. In agreement with ICP analysis, no significant trends on roughness at increasing soaking time can be observed. Thus, this proves that little dissolution of Ca, Ti and Si, happened during the first 24 h.

The chemical composition of implant surfaces profoundly influences cell adhesion, spreading, proliferation, and differentiation [44,45]. Sphene-based substrates better supported hADSCs attachment and proliferation, compared with uncoated cpTi samples, as revealed by the MTT assay. By SEM analysis, a uniform layer of well-spread cells could be observed when cells were cultured onto the sphene-coated surfaces in Osteogenic Differentiation Medium. The results of Alizarin Red S quantification suggested that sphene coating favorably affects the accumulation of mineralized calcium phosphate when cells are cultured in the presence of osteogenic factors. Moreover, cells seeded onto sphene-coated surfaces expressed similar levels of osteoblasts-related genes to those expressed by cells cultured on uncoated cpTi in both culture conditions. We can hypothesize that the combination of the coating and the Osteogenic Differentiation Medium promoted a higher calcium deposition on sphene-coated surfaces than on uncoated cpTi substrates. These findings agree with Wang et al. [43], who demonstrated that sphene glass-ceramic coatings were able to support human osteoblast-like cells (HOBs) attachment, proliferation and differentiation and enhance the expression level of bone-related genes. Furthermore, it was demonstrated that sphene ceramic coatings significantly enhanced HOBs proliferation and ALP activity compared with plasma-sprayed HA coating and uncoated Ti-6Al-4V substrates [14]. Pure dense sphene ceramic disks were found to promote human bone-derived cells attachment and to significantly improve their proliferation and differentiation, as compared with α-CaSiO_3_ ceramic disks [25]. Pure sphene ceramic disks were not only found to modulate HOBs, but also osteoclasts and endothelial cells [26]. As compared with the study of Ramaswamy et al. [26], in the current work, mesenchymal stem cells (MSCs) were preferred to human osteoblast-like cells, human osteoclasts and human microvascular endothelial to in vitro investigate the osteoconductive properties of the biomaterial, since stem cells are the main actors enrolled during the early stages of tissue regeneration. MSCs are stem cells involved on tissue regeneration, which can migrate to the damage site thanks to their receptor and to coordinate all the phase of tissue regeneration [46]. Hypoxia, which usually occurs at the injured sites due to a disruption in blood supply, has been reported to largely contribute to MSC mobilization and homing. The hypoxia-inducible factors (HIFs) are key regulators of the cellular response to decrease in the local oxygen level, also regulating the expression of genes involved in MSC recruitment and migration to the damaged tissues [32,33,34,46,47,48].

In a good agreement with previously published work [14,25,26,43], our results suggest that the ionic environment, determined by the dissolution of Ca and Si ions from the sphene biomaterial, may contribute to stimulate cell proliferation and differentiation. In addition, the in vivo assessment revealed enhanced osseointegration of both sphene-coated and HA-coated Ti-6Al-4V implants, compared to uncoated implants, confirming the in vitro observations and the potential use of sphene coatings in clinical application [26].

## 5. Conclusions

In conclusion, sphene-based crack-free coatings were successfully produced starting from a preceramic polymer and nano-sized fillers and deposited using an automatic airbrush. The coatings presented good chemical stability, as a minimal dissolution occurred only during the first hours of soaking in Tris-HCl solution and then remained stable over the period considered. The coatings also supported hADSCs attachment, proliferation, and differentiation in vitro. Taken together, our findings indicate a potential for use of sphene ceramics as coating materials for orthopedic and dental implants. In vivo studies should be performed to confirm in vitro observations. Further studies are ongoing to increase the reaction efficiency. 

## Figures and Tables

**Table 1:** Linear and surface roughness of as-received cpTi, sphene-coated cpTi, and sphene-coated cpTi after 1 and 7 days of chemical etching in Tris-HCl. Values are expressed as mean (standard deviation).

Samples	Ra (µm)	Rz (µm)	Sa (µm)	Sz (µm)
cpTi	2.82 (0.30)	14.30 (1.38)	3.51 (0.65)	25.15 (5.28)
Sphene	3.94 (0.75)	23.70 (3.74)	3.64 (0.47)	28.09 (3.58)
Sphene Tris-HCl, 1 day	4.66 (0.41)	27.80 (2.05)	4.39 (1.07)	37.28 (6.49)
Sphene Tris-HCl, 3 days	3.66 (0.52)	22.29 (2.85)	4.69 (0.37)	37.70 (6.57)
SpheneTris-HCl,7 days	4.48 (0.37)	27.74 (2.81)	3.99 (0.45)	34.58 (4.96)
